# Gene Deficiency in Activating Fcγ Receptors Influences the Macrophage Phenotypic Balance and Reduces Atherosclerosis in Mice

**DOI:** 10.1371/journal.pone.0066754

**Published:** 2013-06-21

**Authors:** Beñat Mallavia, Ainhoa Oguiza, Oscar Lopez-Franco, Carlota Recio, Guadalupe Ortiz-Muñoz, Iolanda Lazaro, Virginia Lopez-Parra, Jesus Egido, Carmen Gomez-Guerrero

**Affiliations:** 1 Renal and Vascular Inflammation Lab, IIS-Fundacion Jimenez Diaz, Autonoma University, Madrid, Spain; 2 Nephrology Department, IIS-Fundacion Jimenez Diaz, Autonoma University, Madrid, Spain; 3 Institute of Infection, Immunity and Inflammation, University of Glasgow, Glasgow, United Kingdom; 4 Cardiovascular Research Institute, University of California San Francisco, San Francisco, California, United States of America; University of Amsterdam Academic Medical Center, Netherlands

## Abstract

Immunity contributes to arterial inflammation during atherosclerosis. Oxidized low-density lipoproteins induce an autoimmune response characterized by specific antibodies and immune complexes in atherosclerotic patients. We hypothesize that specific Fcγ receptors for IgG constant region participate in atherogenesis by regulating the inflammatory state of lesional macrophages. *In vivo* we examined the role of activating Fcγ receptors in atherosclerosis progression using bone marrow transplantation from mice deficient in γ-chain (the common signaling subunit of activating Fcγ receptors) to hyperlipidemic mice. Hematopoietic deficiency of Fcγ receptors significantly reduced atherosclerotic lesion size, which was associated with decreased number of macrophages and T lymphocytes, and increased T regulatory cell function. Lesions of Fcγ receptor deficient mice exhibited increased plaque stability, as evidenced by higher collagen and smooth muscle cell content and decreased apoptosis. These effects were independent of changes in serum lipids and antibody response to oxidized low-density lipoproteins. Activating Fcγ receptor deficiency reduced pro-inflammatory gene expression, nuclear factor-κB activity, and M1 macrophages at the lesion site, while increasing anti-inflammatory genes and M2 macrophages. The decreased inflammation in the lesions was mirrored by a reduced number of classical inflammatory monocytes in blood. *In vitro*, lack of activating Fcγ receptors attenuated foam cell formation, oxidative stress and pro-inflammatory gene expression, and increased M2-associated genes in murine macrophages. Our study demonstrates that activating Fcγ receptors influence the macrophage phenotypic balance in the artery wall of atherosclerotic mice and suggests that modulation of Fcγ receptor-mediated inflammatory responses could effectively suppress atherosclerosis.

## Introduction

Atherosclerosis is considered not only merely a lipid disorder, but also a chronic inflammatory disease of the arterial wall [1]. Innate (monocyte-derived macrophages) and adaptive (T lymphocytes) immune response both contribute to the arterial inflammation that characterizes atherosclerosis, a dynamic process also involving different cell types such as endothelial cells and vascular smooth muscle cells (SMC). Early atherosclerotic lesions are characterized by the presence of foam cells in the intima as a result of macrophage infiltration and lipid internalization. Foam cells secrete pro-inflammatory cytokines thereby amplifying the initial inflammatory response. Sustained inflammation then results in lesion progression, degradation of the extracellular matrix causing plaque destabilization and rupture [2].

Diversity and plasticity are hallmarks of monocyte/macrophage system, which are reflected in plaque formation and progression [3]. Two major monocyte subsets termed classical (Ly6C^high^) and non-classical (Ly6C^low^) function as control switches of the immune system and maintain the balance between pro- and anti-inflammatory activities [4]. Different macrophage subtypes (M1, classically activated and pro-inflammatory; M2, anti-inflammatory and tissue repair) also participate in inflammatory processes during atherogenesis, being M1 the predominant phenotype in human and murine atheromata. M1 macrophage markers include inducible nitric oxide synthase (NOS), interleukin (IL)-12, tumor necrosis factor-α (TNF-α), interferon-γ (IFN-γ), and arginase (Arg) 2, whereas enhanced expression of IL-4, IL-10, Arg1, mannose receptor and galactose type C-type lectin associate with M2 phenotype [3]–[5].

Antigen-antibody immune complexes (IC) are implicated in many autoimmune and inflammatory diseases. Autoimmunity is also an independent risk factor of atherosclerosis and cardiovascular disease associated mortality [6]. Autoantibodies against oxidized low-density lipoproteins (oxLDL) have been found in blood and lesions of atherosclerotic patients and are considered cardiovascular biomarkers [7], [8]. IgG autoantibodies have a pro-atherogenic effect through the formation of oxLDL-containing IC and cell activation via specific IgG Fc receptors (FcγR) [6], [9]. In mice, four FcγR classes have been identified (FcγRI/CD64, FcγRII/CD32, FcγRIII/CD16, and FcγRIV/CD16-2) differing by their distinct affinity for IgG subclasses, cellular distributions, and effector functions [10], [11]. Activating FcγR (I, III and IV) associate with the common γ-chain that contains the intracellular tyrosine-based activating motif, and upon ligand binding they stimulate phagocytosis, oxidative burst, cytokine release, and antibody-dependent cell-mediated cytotoxicity. In contrast, engagement of inhibitory FcγRIIB abrogates inflammatory responses and instead regulates alternative signaling cascades [10], [11].

Evidence indicates the involvement of IC and FcγR in atherogenesis. Several FcγR have been detected in human atherosclerotic lesions [12], and differential FcγR expression on peripheral blood monocytes associates with severe atherosclerosis [13]–[15]. In hypercholesterolemic mice, gene deficiency in single or multiple FcγR regulates atherogenesis [16]–[19] and renal injury [20]. Accordingly, immunomodulation based on FcγR blockade is proposed as an alternative therapy [21].

It has been previously proven that bone marrow transplantation using knockout mice is a useful approach to study the contribution of hematopoietic cells to atherogenesis, and that hematopoietic-originating macrophages are the major type of cells recruited to the vascular wall during atherosclerotic lesion formation [22]. Accordingly, the present study investigates the effect of activating FcγR deficiency within bone marrow-derived cells on the development of atherosclerotic plaques in the Apolipoprotein E knockout (ApoE^−/−^) mouse model. For this purpose, we used donor marrow from double deficient mice in ApoE and γ-chain [16], the common subunit necessary for assembly, cell-surface localization, and functionality of activating FcγR (types I, III and IV). We therefore hypothesize that functional deficiency in activating FcγR attenuates atherosclerosis progression in mice by reducing the inflammatory state of monocytes/macrophages.

## Methods

### Ethics Statement

The housing and care of animals and all the procedures carried out in this study were strictly in accordance with the Directive 2010/63/EU of the European Parliament and were approved by the Institutional Animal Care and Use Committees of Autonoma University and IIS-Fundacion Jimenez Diaz.

### Bone Marrow Transplantation

Male ApoE^−/−^ (C57BL/6 background, Jackson Laboratory, Bar Harbour, ME) and double deficient mice in γ-chain and ApoE (γ^−/−^ApoE^−/−^) [16] were used in these studies. Donor mice were anesthetized by 2% isoflurane inhalation and sacrificed by cervical dislocation. Bone marrow cells were collected from tibias and femurs of donor mice and treated with Gay’s solution to exclude red blood cell contamination for protection against vascular (thrombotic) injury, as previously described [23]. Recipient mice (ApoE^−/−^, 14-weeks-old) were exposed to 8–9 Gy of radiation and then received 5–10×10^6^ bone marrow cells from donors (ApoE^−/−^ and γ^−/−^ApoE^−/−^) by intravenous injection into the tail vein to generate the respective bone marrow chimeras (A→A, n = 17; γA→A, n = 22). Since donors and transplanted mice had the same genetic background, there were no symptoms of graft-versus-host disease in any of them. Mice were monitored daily for survival (Kaplan-Meier method), and after a 4 week recovery under sterile conditions, they were placed on a Western diet (21.2% fat, 0.15% cholesterol, 17.3% protein; Harlan Labs, Madison, WI) for 10 weeks. Mouse genotype was analyzed by PCR in purified genomic DNA from peripheral blood. Murine γ-chain primer sequences [16], [23]: exon3 (5′-GGAATTCGCTGCCTTTCGGACCTGGAT-3′), exon2 (5′-GGAATTCGATGCTGTCCTGTTTTTGTA-3′) and neo γ-chain in which exon2 was replaced (5′-GCCAACGCTATGTCCTGATAG-3′). Murine ApoE primer sequences [24]: sense21 (5′- GCCTAGCCGAGGGAGGACCG-3′), antisense20 (5′-TGTGACTTGGGAGCTCTGCAGC-3′), and neo19 (5′-GCCGCCCCGACTGCATCT-3′). Wild-type (WT) mice were used as control genotype (γ^+/+^ApoE^+/+^).

### Mouse Sacrifice and Tissue Preparation

Mice were anesthetized by intraperitoneal injection of ketamine (100 mg/kg) and xylazine (15 mg/kg). The adequacy of anesthesia and animal safety were monitored by standard methods (pedal withdrawal, palpebral reflexes, respiration, and skin/mucous membrane color) prior to all experimental procedures. Blood was obtained through retro-orbital puncture. Hearts were perfused with sterile saline via the left ventricle at physiological pressure. Spleens and thoraco-abdominal aortas were preserved in liquid N_2_ and processed for mRNA analysis [25].

### Histological Analysis

Aortic sinus was embedded in OCT and serially cryosectioned (8 µm sections covering ≈1200 µm from valve leaflets). Every third slide was stained with oil-red-O/hematoxylin for morphometric quantification of maximum plaque area [26]. Leukocytes were detected by immunostaining with rat anti-mouse Moma2 (macrophage marker; Serotec, Oxford, UK), CD3 and CD4 (T cell markers; BD Biosciences, Erembodegem, Belgium) antibodies, followed by biotinylated secondary antibody (Amersham, Buckinghamshire, UK), avidin-biotin horseradish peroxidase detection (Dako, Glostrup, Denmark) and hematoxylin counterstain. Macrophage phenotypes and T regulatory cells were analyzed by immunofluorescence using Arg2 (M1 marker; Santa Cruz), Arg1 (M2 marker; Santa Cruz), Mac-3 (total macrophage marker; BD Biosciences), and Forkhead box p3 (Foxp3; BD Biosciences) antibodies, followed by respective secondary antibodies (Alexa Fluor 568 and 488; Invitrogen, Carlsbad, CA) and nuclear counterstain (4′,6-diamidino-2-phenylindole). Proliferating cells and chemokine expression were detected by immunoperoxidase using proliferating cell nuclear antigen (PCNA) and CCL2 antibodies (Santa Cruz Biotechnology, Santa Cruz, CA), respectively. Apoptosis was determined using the Tunel method (ApopTag Peroxidase Kit, Millipore, Billerica, MA). SMC were detected by direct immunofluorescence with α-smooth muscle actin antibody (Cy3 conjugated; Sigma Chemicals, St. Louis, MO). Negative controls were run in parallel with the omission of primary antibodies. Picrosirius red staining was performed for analysis of collagen content by measuring birefringence to plane-polarized light. Activated NF-κB was detected by *in situ* Southwestern histochemistry using digoxigenin-labeled consensus probe, followed by alkaline phosphatase conjugated anti-digoxigenin antibody (Amersham), as described [27]. Positive staining was quantified using Image Pro-Plus 4.5.0.29 (Media Cybernetics, Bethesda, MD) and expressed as percentage of total plaque area or number of cells per lesion area [25], [28].

### Biochemistry and Flow Cytometry

Total serum cholesterol and triglyceride concentrations were measured using standard enzymatic methods. Total serum immunoglobulins were measured by sandwich ELISA using specific antibodies recognizing mouse IgM, IgG, IgG_1_, IgG_2a/c_ and IgG_3_ (BD Biosciences, San Diego, CA). Specific anti-oxLDL immunoglobulins were determined by ELISA using microtitration plates coated with native LDL and oxLDL (1 mg/mL), as previously described [20]. FACS analysis of mouse blood leukocytes was performed with the following fluorochrome-conjugated antibodies and appropriated isotype controls: CD45-FITC, CD3e-PE-Cy7, CD4-PE, CD8a-FITC and CD19-PE form BD Biosciences; CD115-PE and Ly6C-APC from eBioscience (San Diego, CA). Data were acquired using a BD FACS Canto II Flow Cytometer and analyzed with BD FACSDiva software (BD Biosciences).

### Isolation of Murine Macrophages

Peritoneal macrophages were harvested by peritoneal lavage of anesthetized mice (ApoE^−/−^, γ^−/−^ApoE^−/−^ and WT), then plated and allowed to adhere for 2 h. After extensive washing with PBS to eliminate non-attached cells, macrophages were lysed and processed for RNA and protein expression analysis. For foam cell counting, peritoneal macrophages on coverslips were incubated for 6 h in DMEM containing oxLDL alone (25 µg/mL) and oxLDL-containing IC (IgG1 and IgG2 subclasses, 1∶0.5 ratio of oxLDL to IgG), then fixed with 4% paraformaldehyde and stained with oil-red-O/hematoxylin. The total cell and foam cell (>8 lipid droplets) numbers were counted, and the percentage of foam cells was calculated. Bone marrow-derived cells were differentiated for 7 days in DMEM containing 10% FBS, 100 U/mL penicillin, 100 µg/mL streptomycin, 2 mM L-glutamine (Life Technologies, Rockville, MD), and supplemented with 10% L929-cell conditioned medium as a source of macrophage colony stimulating factor. Differentiated macrophages were serum-starved for 12 h before addition of soluble IC (75 µg/mL) for different time periods. The oxLDL-IC and soluble IC used in this study were prepared as previously described [16], [20], [29].

### mRNA Expression Analysis

Total RNA from aorta, spleen and macrophages was extracted with TRIzol reagent (Invitrogen) as described [25], [28]. Expression of target genes was analyzed by real-time quantitative PCR (Applied Biosystem, Foster City, CA) and normalized to housekeeping 18S transcripts.

### Protein Expression Analysis

Macrophages were homogenized in 10 mM Tris-HCl pH 7.4 containing 150 mM NaCl, 1% Triton X-100, 0.5% NP-40, 1 mM EDTA, 1 mM EGTA, 0.2 mM Na_3_VO_4_, 10 mM NaF, 0.2 mM PMSF, and protease inhibitor cocktail. Cytosolic proteins (25 µg) were resolved on SDS-PAGE gels, transferred onto polyvinylidene difluoride membranes and immunoblotted with Arg2 and Arg1 antibodies (0.4 µg/mL) followed by peroxidase-conjugated secondary antibody (Amersham). After visualizing by enhanced chemiluminescence system (Amersham), membranes were reblotted for β-actin (Sigma) as loading control. Immunoblots were quantified using Quantity One software (Bio-Rad, Hercules, CA). CCL2/CCL5 concentrations in macrophage supernatants were analyzed using mouse ELISA kits (R&D Systems, Minneapolis, MN).

### Lucigenin Chemiluminescence Assay

Macrophages were resuspended in 200 µL modified Krebs-Ringer-HEPES buffer (10 mM HEPES pH 7.4, 119 mM NaCl, 4.7 mM KCl, 1.2 mM MgCl_2_, 2.5 mM CaCl_2_, 1.2 mM KH_2_PO_4_, 25 mM NaHCO_3_, and 2 mM glucose), transferred to Röhren tubes and then 5 µM lucigenin and 100 µM NADPH (Sigma) were added. Chemiluminescence was measured with a Luminometer (Berthold Technologies, Bad Wildbad, Germany) by counting the photon emission at 20-s intervals over 10 min. Superoxide production was expressed as relative chemiluminescence units per mg of cell protein [20].

### Statistical Analysis

Data are presented as mean±SEM. Differences across experimental groups were analyzed for statistical significance by unpaired Student’s t test or by one-way ANOVA followed by post-hoc Bonferroni pairwise comparison using GraphPad Prism 5 (GraphPad Software, La Joya, CA). Values of *P*<0.05 were considered significant.

## Results

### Reconstitution with γ^−/−^ApoE^−/−^ Bone Marrow Reduces Atherosclerotic Lesion Size

**Figure 1:**
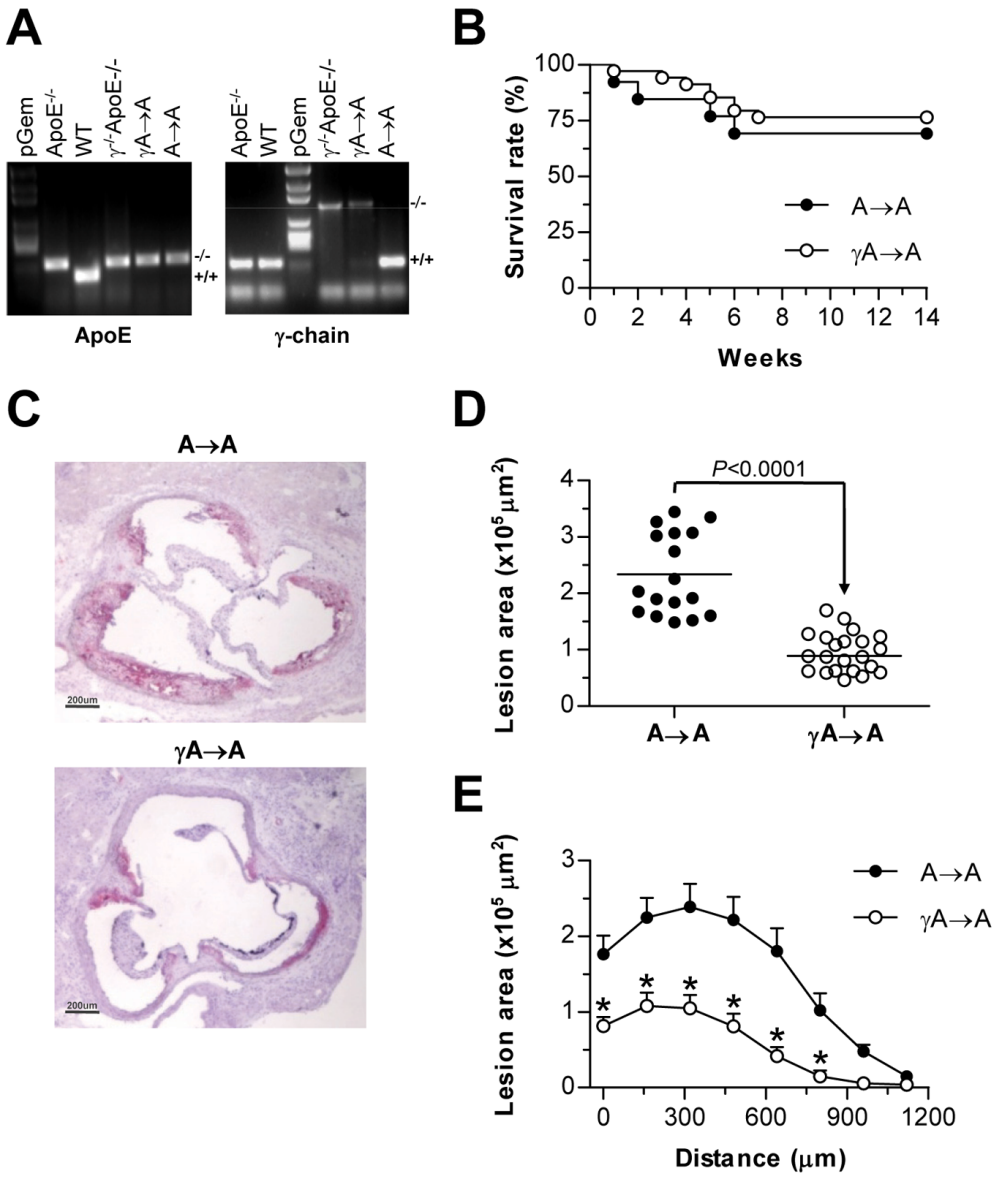
Reconstitution with γ^−/−^ApoE^−/−^ marrow attenuates diet-induced lesion progression in mice. Irradiated ApoE^−/−^ mice were reconstituted with bone marrow from ApoE^−/−^ and γ^−/−^ApoE^−/−^ mice and after 4 weeks were fed on atherogenic diet for 10 weeks. (**A**) PCR analysis of ApoE and γ-chain genes in genomic DNA isolated from donor (ApoE^−/−^ and γ^−/−^ApoE^−/−^), transplanted (A→A and γA→A), and control (WT) mice. (**B**) Kaplan-Meier curves showing survival rate of transplanted mice (log-rank test, *P* = 0.562). (**C**) Representative photomicrographs (magnification x40) of aortic root sections stained with oil-red-O/hematoxylin. (**D**) Quantification of maximum lesion area in the aortic root of A→A (filled circles, n = 17) and γA→A (open circles, n = 22) mice. (**E**) Average of lesion area throughout the studied region in each group. Measurements are expressed as mean±SEM. *P<0.01 versus A→A group.

To examine whether an activating FcγR-dependent mechanism in macrophages accounts for increased atherosclerosis *in vivo*, we performed bone marrow transplantation experiments. Irradiated ApoE^−/−^ mice were reconstituted with bone marrow derived from either γ^−/−^ApoE^−/−^ double deficient mice (γA→A group) or control ApoE^−/−^ donor mice (A→A group), and after recovering were fed on atherogenic diet for 10 weeks. Chimerism in γA→A group was confirmed by genomic PCR analysis of peripheral blood (Figure 1A). Successful engraftment of marrow occurred in more than 90% of animals. After recovery, over 90% of blood cells were from donor origin and blood immune cell composition was similar between genotypes. Furthermore, survival of both chimera groups remained similar along the study (log-rank test, *P*>0.05; Figure 1B).

The size and extent of atherosclerotic lesion was quantified by morphometry after oil-red-O/hematoxylin staining. Aortic lesions in chimeric ApoE^−/−^ recipient mice receiving bone marrow cells from γ^−/−^ApoE^−/−^ donors were 2.5-fold smaller by area (*P*<0.0001) than mice transplanted with ApoE^−/−^ bone marrow (Figure 1, C–E). Moreover, quantification of lipid content in plaques (oil-red-O stained area) revealed a reduced lipid accumulation in γA→A compared with A→A mice (% of plaque area: 16.1±3.1 versus 50.1±12.3; *P* = 0.002; not shown). Both groups had comparable body weight and total cholesterol and triglyceride levels at the end of the study period (Table 1). These findings suggest that FcγRs in bone marrow-derived cells are critical for lesion development in this model.

**Table 1:** Physiologic measurements in transplanted ApoE^−/−^ mice.

Donor→recipient	Body weight (g)		Cholesterol	Triglycerides
combination (n)	Initial	Final	(mg/dL)	(mg/dL)
A→A (17)	18.9±0.2	24.8±0.6	482±11	95±3
γA→A (22)	18.3±0.3	24.2±0.5	475±15	93±5

Reconstitution with bone marrow cells from γ-chain deficient mice did not alter body weight and serum lipid levels. Results (mean±SEM) were compared by two-tailed, unpaired t test with Welch's correction. No significant difference was observed between the experimental groups.

### Attenuated Inflammation and Enhanced Stability in Atherosclerotic Plaques of FcγR Deficient Mice

**Figure 2:**
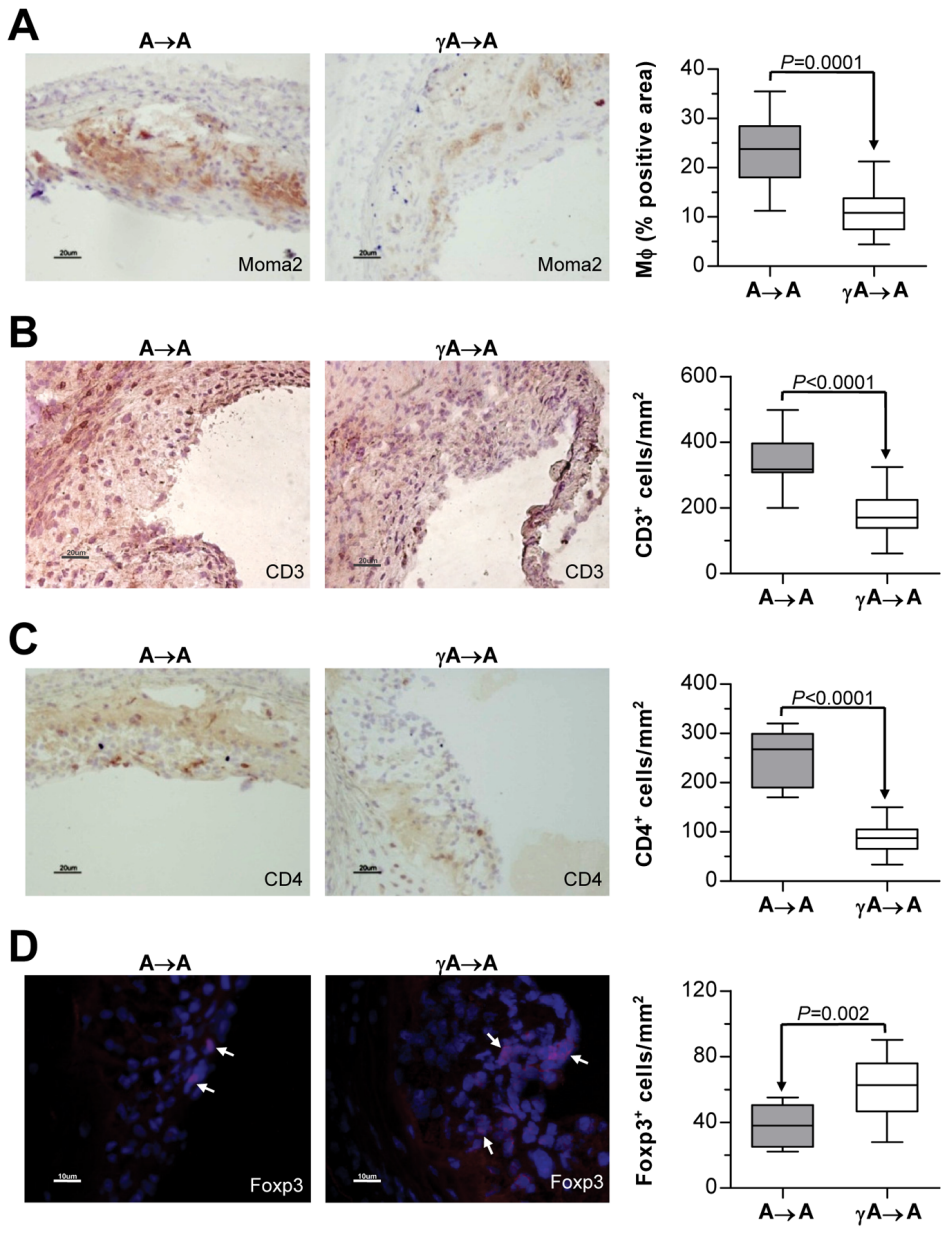
FcγR deficiency alters leukocyte content in atherosclerotic plaques. Histological analysis of macrophages (**A**) and T lymphocytes (**C–D**) in aortic lesions from A→A (n = 17) and γA→A (n = 18) mice. Representative images showing Moma2 (**A**), CD3 (**B**) and CD4 (**C**) immunoperoxidase staining (magnification x200) and Foxp3 (**D**) immunofluorescence (red, with arrows; magnification x400). Quantifications of positive cells per lesion area are indicated in the right panels. Results are expressed as mean±SEM.

Immunohistochemical analysis of cross-sections adjacent to sections of maximum plaque area showed a significantly decreased accumulation of monocytes/macrophages (Moma2^+^) and T lymphocytes (CD3^+^ and CD4^+^) at the atherosclerotic lesions of γA→A mice (Figure 2, A–C), while the number of T regulatory cells (Foxp3^+^) was increased (Figure 2D). Regression analysis showed correlation of lesion area with Moma2, CD3, CD4 and Foxp3 staining in the experimental groups (Pearson r values: 0.87, 0.67, 0.82 and −0.51; P<0.01; not shown), thus indicating a more inflammatory component in larger lesions.

**Figure 3:**
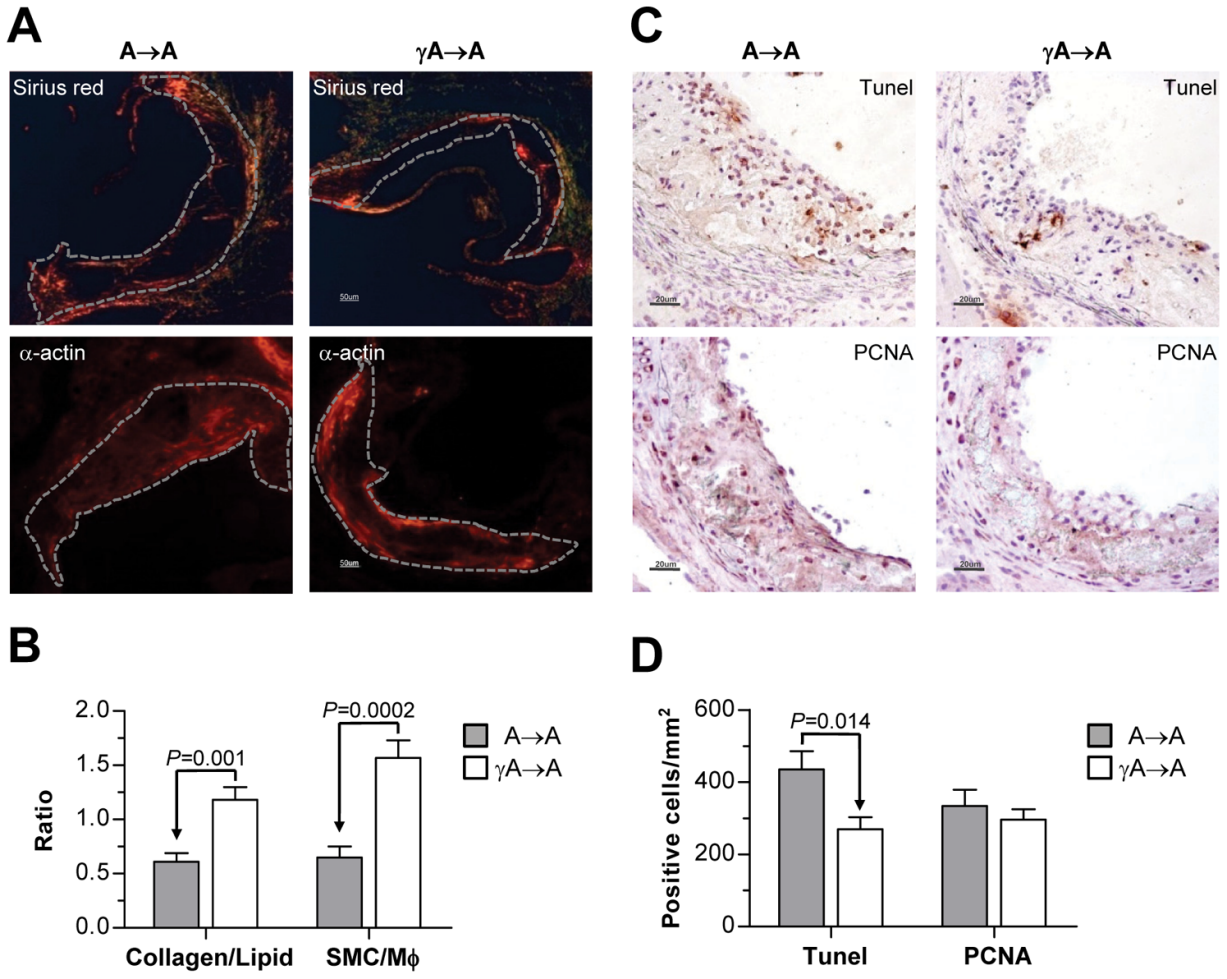
FcγR deficiency increases features of atherosclerotic plaque stability. (**A**) Representative images showing collagen (picrosirius red staining) and SMC (α-actin immunofluorescence) content in aortic sections from transplanted mice. Original magnification x100. Dashed lines highlight the area of atherosclerotic plaque. (**B**) Quantification of collagen to lipid ratio (picrosirius red area/oil-red-O area) and SMC to macrophages (Mφ) ratio (α-actin area/Moma2 area) in A→A and γA→A mice. (**C**) Detection of apoptosis (Tunel staining) and cell proliferation (PCNA staining) in plaques. Magnification x200. (**D**) Quantification of Tunel and PCNA positive cells per lesion area in A→A and γA→A mice. Data are the mean±SEM of n = 12 animals per group.

To assess plaque vulnerability, aortic lesions were analyzed for collagen (picrosirius red staining) and SMC (α-actin immunofluorescence), which are important stabilizing components of the plaque. Atherosclerotic plaques in γA→A mice exhibited a modest, but significant increase in total collagen and SMC content (Figure 3A) as compared with those of control A→A mice (% of total plaque area: picrosirius red, 20.9±1.9 versus 12.2±3.6, *P* = 0.04; α-actin, 18.5±2.1 versus 12.8±1.4, *P* = 0.03). Interestingly, the relative ratio of collagen to lipids (picrosirius red/oil-red-O) and SMC to macrophages (α-actin/Moma2) did markedly increase in the γA→A group (Figure 3B). The higher collagen and SMC content and the lower lipid and leukocyte content in lesions of FcγR deficient mice are consistent with a more stable plaque phenotype. There was also a significant reduction in lesional apoptosis (Tunel staining) in γA→A mice, whereas the number of proliferating cells (PCNA staining) was not different between the two groups (Figure 3, C and D).

### Inflammatory Mediator Expression and Immunoglobulin Isotype Distribution in Transplanted Mice

**Figure 4:**
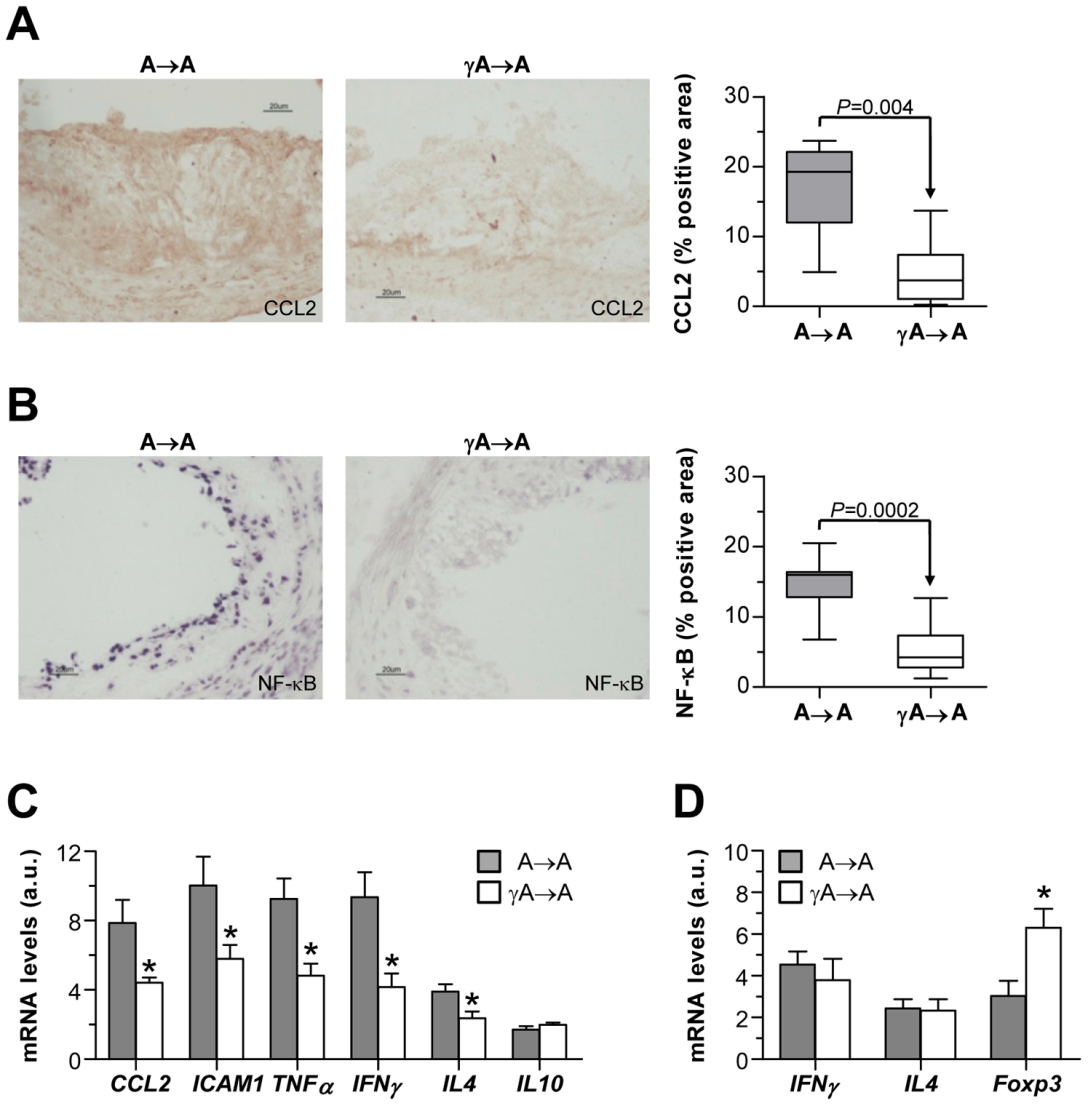
Hematopoietic FcγR deficiency reduces inflammation in atherosclerotic mice. CCL2 protein expression (**A**) and NF-κB nuclear activity (**B**) in aortic sections from transplanted mice. Representative micrographs (magnification x200) and quantification of positive staining per lesion area are shown. Gene expression levels in aorta (**C**) and spleen (**D**) of A→A and γA→A mice were measured by real-time PCR. Values were normalized to 18S and expressed as arbitrary units (a.u.). Data are mean±SEM of total number of animals per group. *P<0.03 versus A→A group.

Lesions of γA→A mice showed significantly less protein expression of macrophage chemokine CCL2 than controls (Figure 4A). Likewise, Southwestern histochemistry showed an attenuated activity of NF-κB, the transcription factor that regulates many pro-inflammatory and pro-atherosclerotic genes (Figure 4B). Levels of CCL2 and NF-κB correlate with lesion size (Pearson r = 0.91 and 0.85; *P*<0.0001; not shown), and macrophage content (Pearson r = 0.78 and 0.74; *P*<0.0001; not shown) in the experimental groups.

Quantitative real-time PCR analysis on RNA from aortic tissues revealed considerable differences in the gene expression of chemokine CCL2 and the intracellular adhesion molecule-1 (ICAM-1) in γA→A mice (% decrease versus control A→A group: 44±4 and 42±8, respectively; *P*<0.04; Figure 4C). FcγR deficiency also significantly reduced the aortic expression levels of the signature cytokines for Th1 (TNF-α and IFN-γ) and Th2 (IL-4) responses (% decrease: 48±8, 55±8 and 40±10, respectively; Figure 4C), although their relative proportion did not change between A→A and γA→A groups (TNF-α/IL-4 ratio: 2.3±0.3 versus 2.1±0.3; IFN-γ/IL-4 ratio: 2.1±0.4 versus 1.9±0.3; *P*>0.05). Analysis of spleen samples demonstrated similar expression of Th1/Th2 cytokines (IFN-γ/IL-4 ratio: A→A, 2.1±0.4; γA→A 1.9±0.3; *P*>0.05), and overexpression of T regulatory cell-associated transcription factor Foxp3 in FcγR deficient mice (Figure 4D).

**Figure 5:**
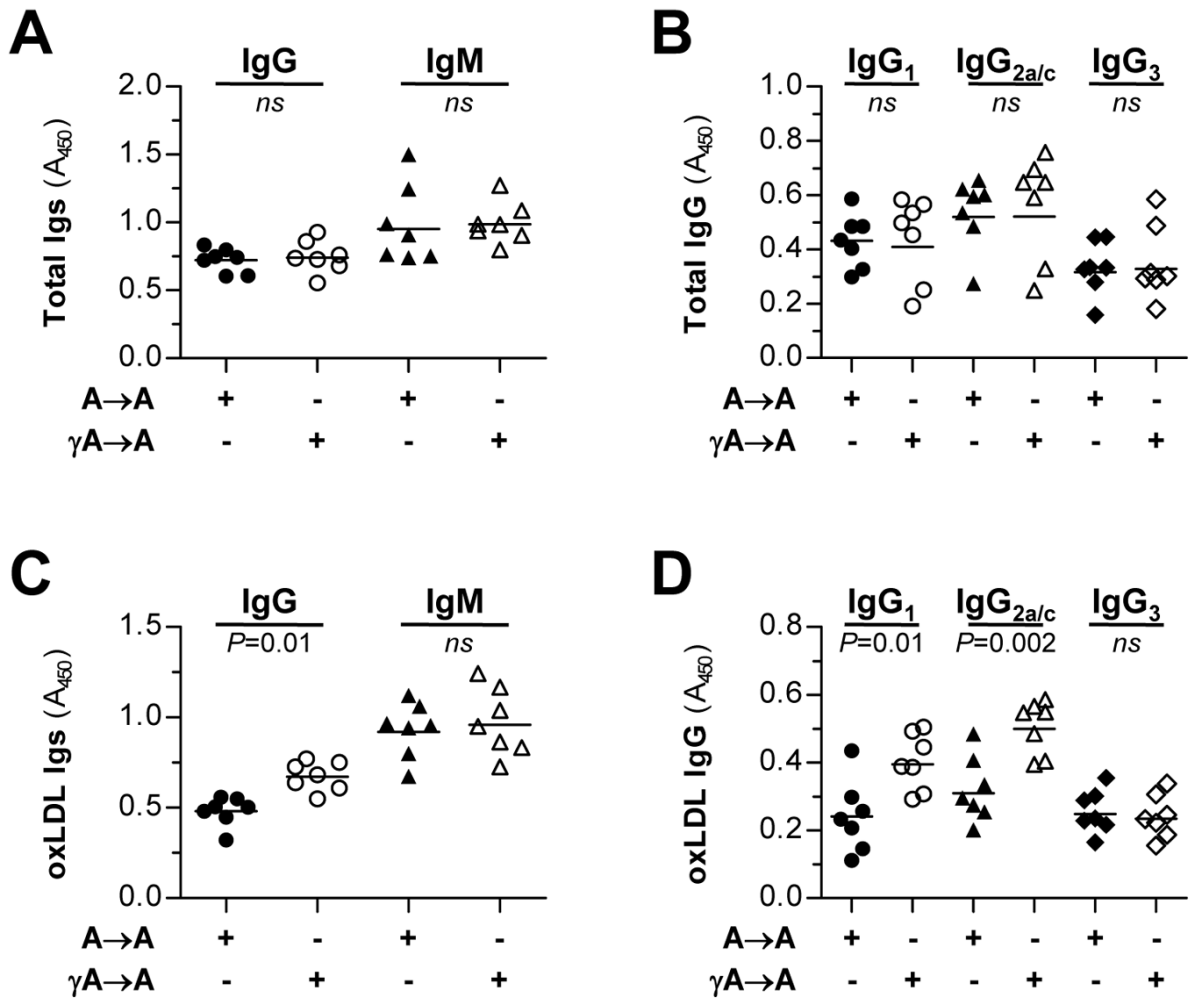
Immunoglobulin isotype distribution in transplanted mice. Sera from A→A (filled symbols, n = 7) and γA→A (open symbols, n = 7) transplanted mice were analyzed for total (**A, B**) and oxLDL-specific autoantibodies (**C, D**). IgG and IgM isotypes (**A, C**) and IgG subclasses (**B, D**) were determined by ELISA using specific antibodies as indicated in Methods. Values are expressed as absorbance units at λ = 450 nm.

Changes in the adaptive immune response were assessed by measuring serum antibody titers to oxLDL epitopes. Total antibody isotypes (IgM and IgG) and IgG subclasses (1, 2a/c and 3) were not different between the experimental groups (Figure 5, A and B). Interestingly, oxLDL-specific IgG response, but not IgM, was increased in γA→A mice relative to A→A controls (Figure 5C). Determination of anti-oxLDL IgG subclasses revealed significant differences in IgG_1_ and IgG_2a/c_ levels in γA→A mice (Figure 5D). However, the relative ratio of IgG_2a/c_ (Th1 response) to IgG_1_ (Th2 response) did not change between the groups (1.2±0.1 versus 1.3±0.1; *P*>0.05), thus indicating no apparent Th1/Th2 bias.

### FcγR Deficiency Affects Monocyte/macrophage Activation State in Atherosclerotic Mice

Our findings thus far indicate that FcγR deficiency reduced the accumulation of inflammatory cells in atherosclerotic plaque. We then examined whether FcγR deficiency influenced the leukocyte count or subset frequency in peripheral blood using flow cytometry (Table 2). Blood counts for total leukocyte number and subgroups (B cells, T cells and monocytes) revealed no differences between A→A and γA→A mice. Interestingly, analysis of monocyte subset distribution (Ly6C^high^ and Ly6C^low^) of CD115^+^-gated monocytes revealed decreased percentages of Ly6C^high^ and increased percentages of Ly6C^low^ subsets in γA→A group. This resulted in a significant decrease in the ratio of Ly6C^high^:Ly6C^low^ monocytes in FcγR deficient mice compared with controls (Table 2), thus indicating a reduced activation state of circulating monocytes.

**Table 2:** Flow cytometry of blood leukocyte populations in mice.

Cell type	Marker	A→A	γA→A
Leukocytes	CD45	82.6±10.3	80.0±7.0
T lymphocytes	CD3	40.0±4.9	46.1±7.8
	CD4	15.6±2.5	19.3±6.7
	CD8	4.6±1.2	5.9±1.9
B lymphocytes	CD19	33.9±5.7	31.9±7.2
Monocytes	CD115	17.6±2.5	18.1±5.0
	Ly6C^high^:Ly6C^low^	4.8±0.3	2.4±0.2*

Quantitative analysis shows significant decrease in the ratio of Ly6C^high^:Ly6C^low^ among CD115^+^ monocytes of FcγR deficient mice. Numbers indicating percentage of positive cells are means ± SEM of 7 animals. *P = 0.03 versus A→A.

**Figure 6:**
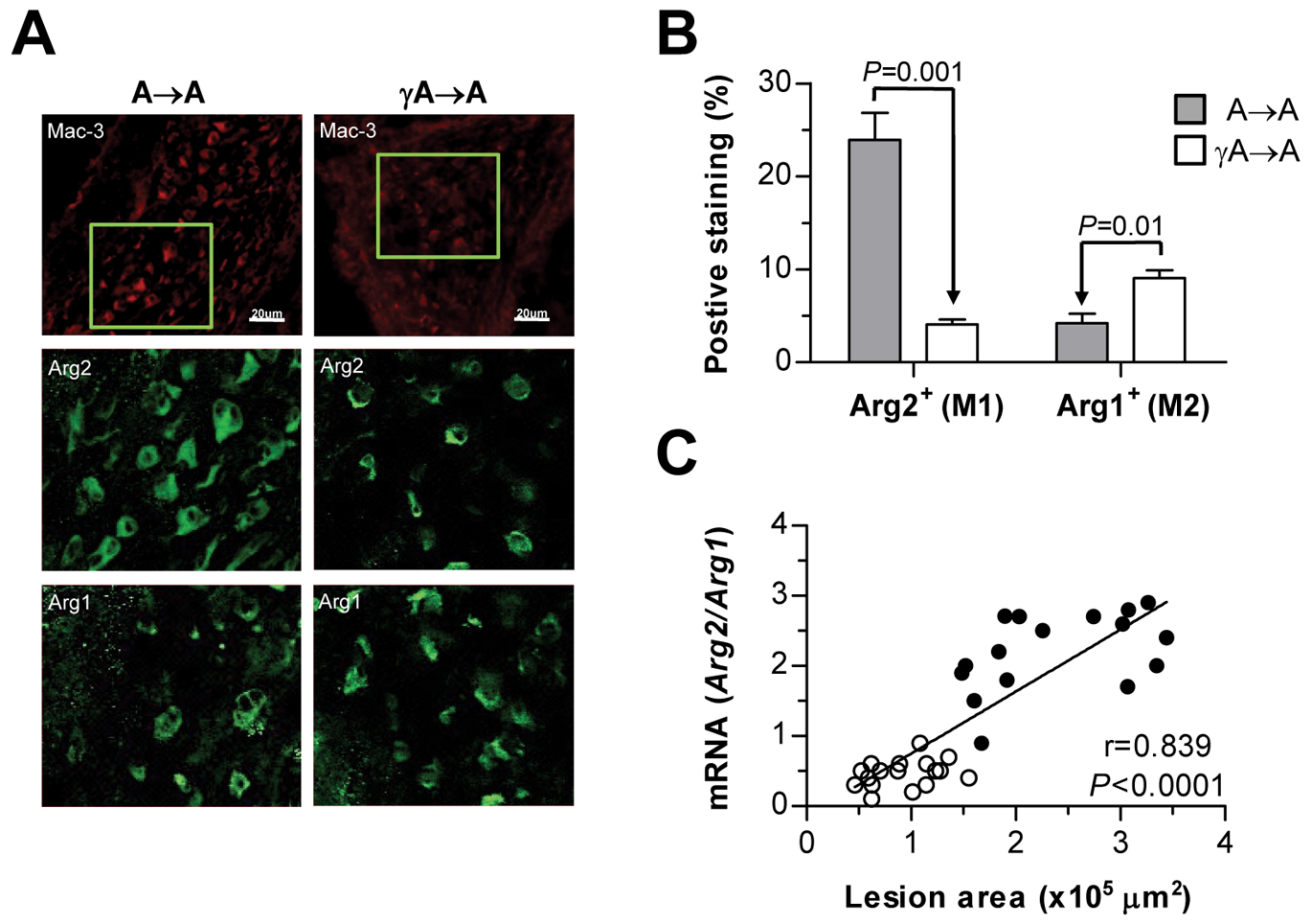
Effect of FcγR deficiency on macrophage phenotype in atherosclerotic lesions. (**A**) Immunofluorescence analysis of total macrophages (Mac-3; red), and phenotypes M1 and M2 (Arg2 and Arg1, respectively; green) in aortic sections from transplanted mice. Representative photomicrographs (top panel, magnification x200) and high-magnification images (x400) of the selected regions (squares). (**B**) Quantification of Arg2^+^ and Arg1^+^ macrophages per lesion area. Values represent the mean±SEM of total number of animals per group. (**C**) Real-time PCR analysis of Arg isoforms in aortic tissue. Correlation between Arg2/Arg1 expression and lesion area in A→A (filled circles, n = 16) and γA→A (open circles, n = 17) mice is shown.

To further examine the macrophage phenotype associated with FcγR deficiency in atherosclerotic lesions, the gene expression of Arg2 and Arg1 isoforms was analyzed to distinguish between the pro-inflammatory classical M1 and the anti-inflammatory alternative M2 response, respectively. Immunofluorescence and gene expression analysis (Figure 6) revealed that both macrophage markers are present in atherosclerotic lesions, being Arg2 (M1) most abundantly expressed in A→A mice, and Arg1 (M2) the predominant marker in γA→A. Furthermore, calculation of Arg2/Arg1 ratio (average per group: A→A, 2.2±0.1; γA→A, 0.5±0.1, *P*<0.0001) and regression analysis (Figure 6C) demonstrated that higher lesion size correlated with the dominance of M1 over the M2 macrophage phenotype marker.

### FcγR Deficiency Prevents Foam Cell Formation and Inflammatory Responses in Macrophages

**Figure 7:**
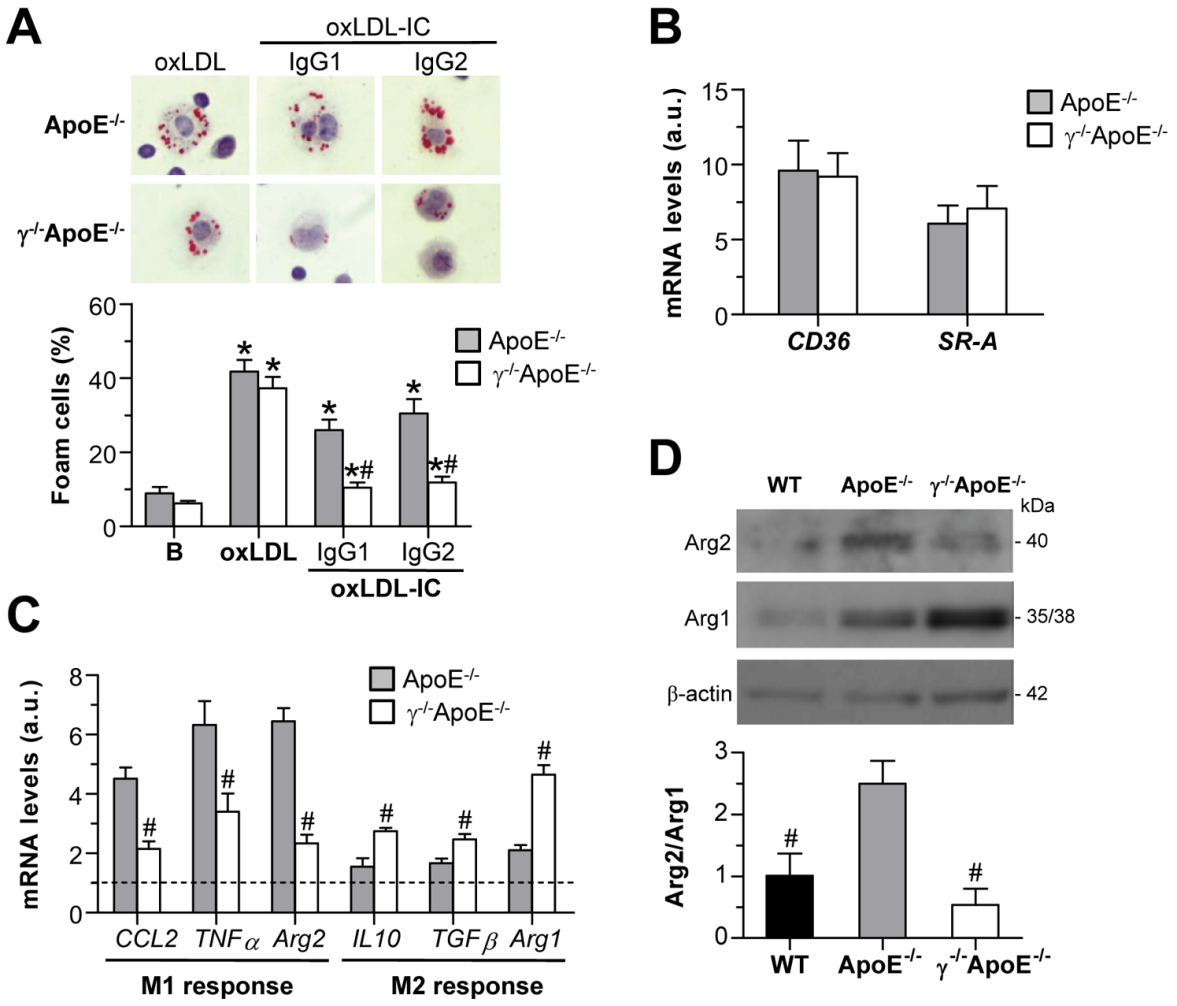
FcγR deficiency inhibits foam cell formation and pro-inflammatory gene expression in peritoneal macrophages. (**A**) Macrophages from ApoE^−/−^ and γ^−/−^ApoE^−/−^ mice were exposed to oxLDL alone (25 µg/mL) and oxLDL-IC (IgG1 and IgG2, 1∶0.5 ratio) for 6 h. Cells containing oil-red-O stained fat droplets were considered foam cells. Representative images (original magnification x400) and macrophage foam cell quantification are shown. (**B**) Real-time PCR analysis of scavenger receptors (CD36 and SR-A) in freshly isolated peritoneal macrophages. Values corrected by 18S expression are expressed as arbitrary units (a.u.). (**C**) Expression levels of M1 and M2 response genes in macrophages from WT, ApoE^−/−^ and γ^−/−^ApoE^−/−^ mice. Data are normalized to WT values ( = 1; dashed line). (**D**) Western blot analysis of Arg isoforms in peritoneal macrophages from WT, ApoE^−/−^ and γ^−/−^ApoE^−/−^ mice. Data of densitometry are corrected by loading control (β-actin) and expressed as Arg2/Arg1 ratio. Bars represent the mean±SEM of n = 5–7 animals per group. *P<0.05 versus Basal; # *P*<0.05 versus ApoE^−/−^ cells.

Studies in peritoneal macrophages were further performed to assess lipid accumulation and foam cell formation in atherosclerotic mice. Quantification of oil-red-O staining in resident peritoneal cells from mice on high-fat diet revealed an increased number of foam cells in ApoE^−/−^ as compared with WT littermates (% macrophage foam cells: WT, 26.1±1.9; ApoE^−/−^, 61.8±6.4; *P* = 0.01; n = 6) and a decrease in FcγR deficient mice (% macrophage foam cells: 33.0±5.3, *P* = 0.01 versus ApoE^−/−^; n = 6). In other experiments, peritoneal macrophages from ApoE^−/−^ and γ^−/−^ApoE^−/−^ mice on chow diet were incubated for 6 h in the presence of oxLDL alone and oxLDL-containing IC. As shown in Figure 7A, foam cell formation was similarly induced by oxLDL in both cell types. By contrast, foam cell numbers after oxLDL-IC incubation were significantly reduced in peritoneal cells from γ^−/−^ApoE^−/−^ mice versus ApoE^−/−^ mice (Figure 7A), despite having equivalent expression levels of scavenger receptors (SR-A and CD36; Figure 7B). These results confirm the involvement of FcγR in oxLDL-IC uptake and foam cell formation.

Peritoneal macrophages from FcγR deficient mice exhibited a reduced expression of pro-inflammatory genes (CCL2, TNF-α and Arg2) and increased expression of M2 response genes (IL-10, TGF-β and Arg1) (Figure 7C). Accordingly, analysis of Arg2 and Arg1 protein expression revealed that the relative ratio of M1 to M2 markers was higher in peritoneal macrophages from ApoE^−/−^ mice as compared with WT littermates, and was significantly reduced by FcγR deficiency (Figure 7D).

**Figure 8:**
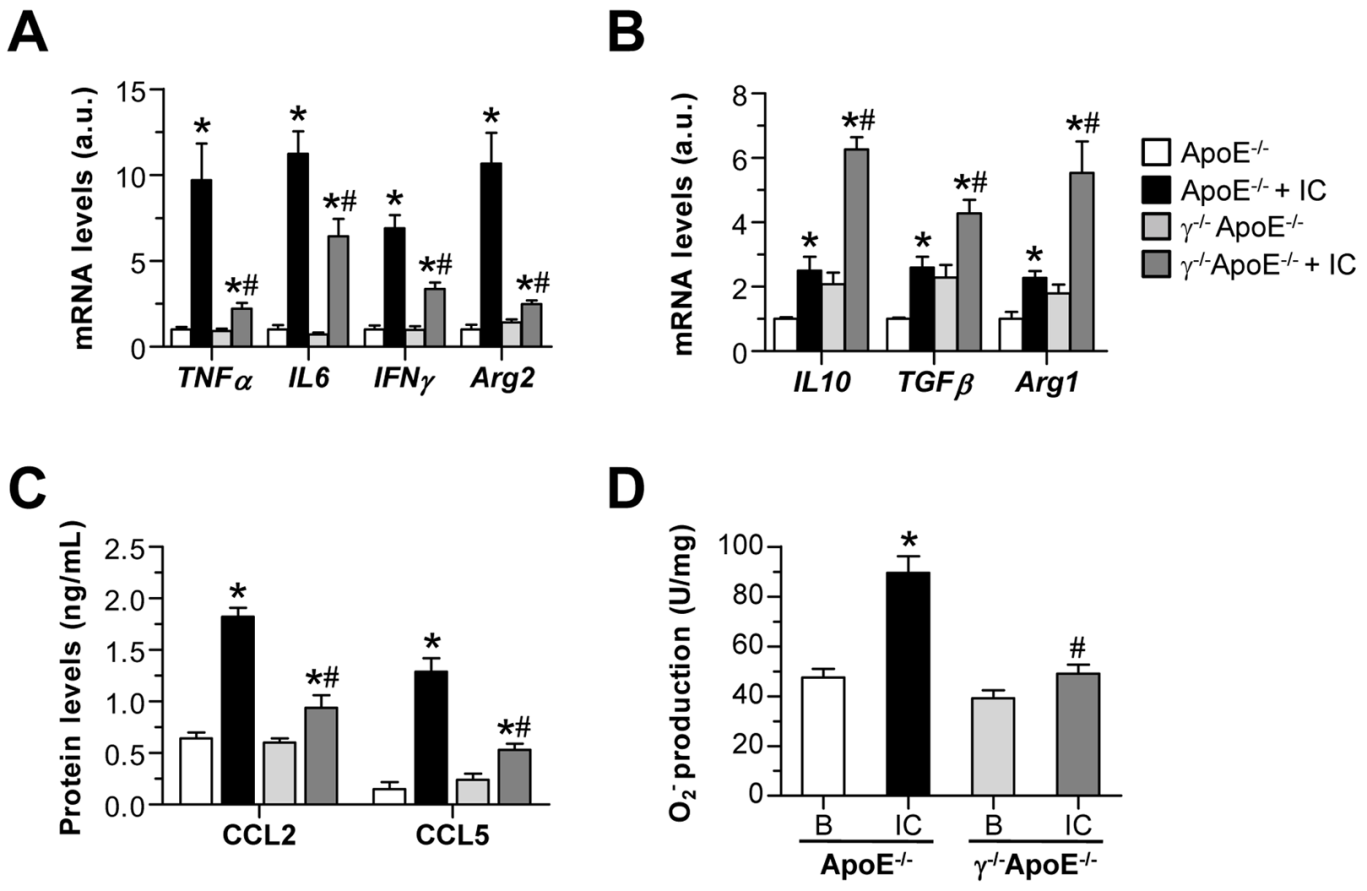
Responses of bone marrow-derived macrophages to FcγR stimulation. Bone marrow-derived macrophages from ApoE^−/−^ and γ^−/−^ApoE^−/−^ mice were stimulated with soluble IC (75 µg/mL). Real-time PCR analysis of M1 (**A**) and M2 (**B**) response genes at 6 h of stimulation. Expression data are normalized to control ApoE^−/−^ values ( = 1) and displayed as arbitrary units (a.u.). (**C**) Chemokine production in supernatants was detected by ELISA. (**D**) NADPH oxidase-dependent superoxide anion production (lucigenin assay) at 1 h of stimulation. Values are expressed as chemiluminescence units per mg of cell protein. Data represent the mean±SEM of 4–6 independent experiments.*P<0.05 versus basal conditions; ^#^ *P*<0.05 versus ApoE^−/−^ cells.

Further experiments were performed in bone-marrow derived mouse macrophages. Treatment with soluble IC resulted in a significant polarization of ApoE^−/−^ macrophages to the M1-like phenotype, as evidenced by a predominant Arg2 levels (Figure 8A) with concomitant increase in other classical activation markers, including pro-inflammatory cytokines (TNF-α, IL-6 and IFN-γ; Figure 8A) and chemokines (CCL2 and CCL5; Figure 8C). In contrast, IC failed to prime γ^−/−^ApoE^−/−^ macrophages to the pro-inflammatory phenotype, as evidenced by reduced expression levels of M1 markers (Figure 8, A and C) and increased M2 response genes (Figure 8B). Furthermore, macrophages from FcγR deficient mice exhibited reduced oxidative stress in response to IC stimulation, as measured by NADPH oxidase-dependent superoxide production (Figure 8D).

## Discussion

The present study demonstrates a key role for macrophage FcγR in initiation and progression of vascular lesions in experimental atherosclerosis. Our bone marrow transplantation studies show that hematopoietic gene deficiency in γ-chain, the common signaling subunit of activating FcγR (types I, III and IV), is sufficient to limit atheroma plaque formation in mice. The underlying mechanisms are independent of serum lipids and Th1/Th2 changes. Instead, our data indicate that FcγR deficiency reduces circulating inflammatory monocytes, impairs foam cell formation and skews the distribution of macrophage population within the plaque towards the anti-inflammatory phenotype.

Inflammation is recognized as a major contributor to atherogenesis through adverse effects on lipoprotein metabolism and arterial wall biology. Accumulating evidence indicates that both humoral and cell-mediated pro-atherogenic immunity against altered self-antigens generated by hypercholesterolemia (such as oxLDL particles) play a predominant role in the atherogenic process [6]. Clinical studies have demonstrated a positive association between the degree of established atherosclerosis and antibody levels to oxLDL, mainly IgM and IgG isotypes [7], [8]. High production of IgM and IgG anti-oxLDL antibodies has also been observed in hypercholesterolemic mice [30], [31]. It has been proposed that atheroprotective IgM contributes to maintain low plasma levels of oxLDL and blocks the uptake of oxLDL by lesional macrophages, thereby preventing the oxLDL accumulation and foam cell formation [32], [33]. On the other hand, IgG antibodies are pro-atherogenic through the formation of oxLDL-containing IC and induction of FcγR-mediated inflammatory responses in resident and infiltrating cells [6], [34]. In fact, FcγR is the most important macrophage receptor involved in the clearance of oxLDL-IC [9]. In the current study, despite the difference in atherosclerosis severity, the IgM anti-oxLDL response remained unchanged in mice reconstituted with γ^−/−^ApoE^−/−^ bone marrow. Interestingly, the IgG antibodies to oxLDL increased, probably due to impaired phagocytic activity in FcγR deficient cells. In mouse models, severe hypercholesterolemia is associated with an antibody switch from Th1 (IgG_2a_) to Th2 (IgG_1_) response [30], whereas B cell depletion reduces the production of anti-oxLDL antibodies and limits atherosclerosis development [35]. As the concentrations of both IgG subclasses were enhanced in the serum of γ^−/−^ApoE^−/−^ transplanted mice, we can consider that, instead of a polyclonal activation of B cells, the two Th1- and Th2-type T cells are indeed involved in the antibody response against oxLDL. Although this antibody pattern suggests a more pronounced Th1 and Th2 immune response in FcγR deficient mice, our analysis of the Th1/Th2 ratio did not substantiate systemic changes in Th cell composition. Furthermore, flow cytometry analysis revealed similar content of blood leukocytes and subgroups (B cells, T cells and monocytes) in both groups of transplanted mice. Interestingly, lack of FcγR declined the number of circulating Ly6C^high^ monocytes, the classical inflammatory subset predominant in hypercholesterolemic mice [36] that is preferentially adhered to activated endothelium, accumulated in lesions, and locally differentiated into macrophages [4], [37]. Our observations confirm these previous reports highlighting the prominent role of classical monocytes in atherogenesis and as source for lesional macrophages [36], [37], and suggest that a reduced monocyte activation state is involved in the atheroprotective effect of FcγR deficiency.

The coordinate expression of activating and inhibitory FcγR ensures the homeostasis of IC-driven inflammatory responses. In fact, gene deficiency in activating FcγR is associated with diminished inflammation in response to IC, while severe inflammation was observed following deletion of inhibitory FcγRIIB [11], [38]. In atherosclerotic mice, FcγRIII deficiency caused a reduction in aortic lesion size [17], whereas FcγRIIB deletion aggravates the atherosclerotic process [18]. Our early study demonstrated that mice lacking γ-chain associated FcγR developed less atherosclerotic plaques characterized by a reduction in leukocyte content and inflammatory mediators [16]. Recently, Ng *et al.* [19] reported an increased generation of circulating T regulatory cells with a concomitant decrease in Th17 cells in γ-chain deficient mice. The current data complement these previous immunological changes and also directly address the functional role of FcγR activation on resident and recruited monocytes/macrophages during atherosclerosis promotion. We cannot, however, rule out that FcγR on other cells also modulate plaque development and progression, since bone marrow transplantation reconstitutes more than monocytes/macrophages [22]. In fact, hematopoietic stem cells are able to differentiate into endothelial and vascular SMC progenitors [39], and bone marrow cells accumulated in atherosclerotic lesion differentiate into vascular SMC and foam cells thereby contributing to vascular remodeling [40]. Moreover, different FcγR are found expressed on endothelial and SMC, which demonstrate pro-atherogenic response to FcγR stimulation *in vitro* [16], [28], [41]. Thus, further studies will be needed to demonstrate conclusively the relative contribution of FcγR on both resident vascular and infiltrating cells in this process.

The presence of oxLDL antibodies and FcγR in atheroma plaques has been clearly demonstrated in patients [12], [42]. In mice, adoptive transfer studies of CD4^+^ T cells specific to oxLDL have shown that Th1 cell responses play an important role in promoting atherosclerosis [43]. Our study demonstrates that reconstitution with γ-chain deficient marrow in mice with developing atherosclerosis significantly attenuates further progression without affecting plasma lipid levels. First, the decrease in atheroma size correlates with a reduced number of T cells and macrophage foam cells and higher T regulatory cell content in aortic lesions. This indicates a deficient pro-atherogenic immune activation in the artery and also a key role of T regulatory cell function in FcγR deficient mice, consistent with previous studies of the protective effect of T regulatory cells in atherosclerosis progression [19], [44]. Secondly, hematopoietic FcγR deficiency resulted in the development of a lipid-poor collagen-rich atherosclerotic lesion characterized by higher collagen/lipid and SMC/macrophage ratios and low apoptosis, thereby suggesting a more stable plaque phenotype. Because most acute complications of atherosclerosis are caused by the rupture of an unstable lipid-rich collagen-poor lesion [1], strategies to modulate activating FcγR may have a beneficial role in slowing lesion progression.

In parallel with the reduction in leukocyte infiltration, hematopoietic FcγR deficiency leads to reduced local expression of genes involved in leukocyte activation and migration during atherogenesis [45], [46], including adhesion molecule ICAM-1, chemokine CCL2, pro-atherogenic Th1 cytokines TNF-α and IFN-γ, and Th2 cytokine IL-4. However, and consistent with the IgG subclass ratios, the relative proportion of Th1/Th2 cytokine expression in aorta and spleen remained unmodified, thus discarding either lesional or systemic Th1/Th2 imbalance.

Transcription factor NF-κB is a major regulator of immunoinflammatory responses that controls cytokine gene expression and has a pivotal role in atherogenesis [46]. We have earlier demonstrated NF-κB activation and target gene expression in experimental atherosclerosis [25] and also in cultured cells after FcγR cross-linking [16], [20], [29]. Keeping in line with this, our study shows that FcγR deficiency blunted the activity of NF-κB in cells of the intima and media, thus indicating that NF-κB’s effect on inflammation and lipid handling can be regulated by IC in vascular and infiltrating cells. These data do not exclude a contribution of systemic inflammatory factors, such as serum cytokines and C-reactive protein previously shown to be increased in atherosclerosis [45], [47], but suggest that the atherogenic effects of FcγR are mainly mediated via inflamed cells localized in plaques.

Besides effects on the plaque macrophage content, FcγR deficiency had a dramatic effect on the inflammatory state of these cells, with a general decrease and increase, respectively, in markers of M1 (classically activated) and M2 (alternatively activated or tissue repair) macrophages. Although this classification scheme has been considered to be overly simplistic, it has been a useful start with which to broadly characterize the macrophage heterogeneity known to occur in many tissues including atherosclerotic plaques [3]. Previous works have evaluated the phenotype of macrophages associated with progression of atherosclerosis in mice. While early lesions are infiltrated by reparative M2 macrophages, the M1 sub-type appeared and prevailed during plaque progression [48]. These M1 cells accumulated in damaged vessel wall are able to secrete many inflammatory cytokines that amplify Th1 immune response, affect SMC function and proliferation, and also contribute to lipoprotein oxidation through the production of reactive oxygen species [4], [49]. In our work, the reduction in plaque lipid content and lesion size was accompanied by a decrease in infiltrating macrophages and pro-inflammatory mediators, along with a lower ratio of M1/M2, as determined by differential expression of Arg isoforms. Macrophage Arg converts arginine to ornithine and urea and competes directly for substrate with NOS, and the two isoforms (mitochondrial Arg2 and cytosolic Arg1) have been evaluated as surrogate markers of M1 and M2 phenotypes during atherosclerotic progression [48], [50]. Indeed, classical M1 macrophages in the lesions express Arg2 and inducible NOS, but not Arg1, while alternatively activated M2 macrophages exclusively express Arg1. In our paper, the prevalence of M2 marker (Arg1) in aortic lesions from FcγR deficient mice suggests that these smaller lesions contain macrophages of anti-inflammatory and tissue repair phenotype and consequently may be more stable. Concurrently, the pattern of pro- and anti-inflammatory cytokine expression in isolated macrophages strongly indicates that FcγR deficient macrophages resemble features of anti-inflammatory M2-like macrophages. It has been previously reported that IC-stimulated macrophages more closely resemble M1 than M2 in terms of cytokine profile and functionality [51]. Furthermore, selective cross-linking of inhibitory FcγRII/CD32, but not activating FcγR (CD64 and CD16) accounts for the induction of CCL1 chemokine in human monocytes and is linked to M2 activation program [52]. An additional mechanistic study [53] revealed the involvement of activating FcγR and NF-κB pathway in the polarization of human monocytes toward pro-inflammatory M1 macrophages by C-reactive protein. In line with this, our data indicate that the pro-atherogenic role of activating FcγR is related to the pro-inflammatory and pro-oxidative action on macrophages.

Collectively, our results document a role for activating FcγR in controlling the macrophage phenotypic balance in the artery wall of atherosclerotic mice. We therefore propose that strategies to modulate FcγR activating/inhibitory balance and effector functions could suppress atherosclerosis by regulating macrophage inflammatory states into the reparative M2 phenotype.
